# Strong Convergence Theorems for a Common Fixed Point of a Finite Family of Bregman Weak Relativity Nonexpansive Mappings in Reflexive Banach Spaces

**DOI:** 10.1155/2014/493450

**Published:** 2014-03-13

**Authors:** Habtu Zegeye, Naseer Shahzad

**Affiliations:** ^1^Department of Mathematics, University of Botswana, Private Bag 00704, Gaborone, Botswana; ^2^Department of Mathematics, King Abdulaziz University, P.O. Box 80203, Jeddah 21589, Saudi Arabia

## Abstract

We introduce an iterative process for finding an element of a common fixed point of a finite family of Bregman weak relatively nonexpansive mappings. Our theorems improve and unify most of the results that have been proved for this important class of nonlinear operators.

## 1. Introduction

Throughout this paper, *f* : *E* → (−*∞*, +*∞*] is a proper, lower semicontinuous, and convex function, where *E* is a real reflexive Banach space with E* as its dual. Denote the domain of *f* by dom⁡*f*; that is, dom⁡*f* = {*x* ∈ *E* : *f*(*x*) < *∞*}. Let *f* : *E* → (−*∞*, +*∞*] be a* Gâteaux differentiable* (see Section 2) function. The function *D*_*f*_ : dom⁡*f* × int(dom*f*)→[0, +*∞*) defined by

(1) $$\begin{array}{c} D_{f}\left(x,y\right):=f\left(x\right)-f\left(y\right)-\langle \nabla f\left(y\right),x-y\rangle , \end{array}$$

where ∇*f* is the gradient of *f*, is called the* Bregman distance with respect to f* [1]. The following property of Bregman distance function is known:

(2) Df(x,z)−Df(x,y)−Df(y,z)  =〈∇f(y)−∇f(z),x−y〉,

for *x* ∈ dom⁡*f* and *y*, *z* ∈ int(dom*f*) (see [1, 2]).

A* Bregman projection* with respect to *f* [1] of *x* ∈ int(dom*f*) onto the nonempty closed and convex set *C* ⊂ dom⁡*f* is the unique vector *P*_*C*_^*f*^(*x*) ∈ *C* satisfying

(3) $$\begin{array}{c} D_{f}\left(P_{C}^{f}\left(x\right),x\right)=\inf\left\{D_{f}\left(y,x\right):y\in C\right\}. \end{array}$$

**Remark 1**

If *E* is a smooth Banach space, setting *f*(*x*) = ||*x*||^2^, for all *x* ∈ *E* we have ∇*f*(*x*) = 2*Jx*, for all *x* ∈ *E*, where *J* is the normalized duality mapping from *E* onto E*, and hence we have the following.

(i) *D*_*f*_(*x*, *y*) reduces to *D*_*f*_(*x*, *y*) = ||*x*||^2^ − 2〈*x*, *Jy*〉+||*y*||^2^ = *ϕ*(*x*, *y*), for all *x*, *y* ∈ *E*, which is the Lyapunov function introduced by Alber [3]. If *E* = *H*, a Hilbert space, *J* is identity mapping and hence *D*_*f*_(*x*, *y*) becomes *D*_*f*_(*x*, *y*) = ||*x*−*y*||^2^, for *x*, *y* ∈ *H*.
(ii) The Bregman projection *P*_*C*_^*f*^(*x*) reduces to the generalized projection Π_*C*_(*x*) (see, e.g., [3]) which is defined by
  (4) $$\begin{array}{c} \phi \left(\Pi_{C}\left(x\right),x\right)=\underset{y\in C}{\min}\;\phi \left(y,x\right). \end{array}$$

Let *C* be a nonempty and convex subset of int(dom*f*) and let *T* : *C* → int(dom*f*) be a mapping. A mapping *T* : *C* → *C* is said to be* nonexpansive* if ||*Tx* − *Ty*|| ≤ ||*x* − *y*||, for all *x*, *y* ∈ *C*. *T* is said to be* quasi-nonexpansive* if *F*(*T*) ≠ *∅* and ||*Tx* − *p*|| ≤ ||*x* − *p*||, for all *x* ∈ *C* and *p* ∈ *F*(*T*), where *F*(*T*) stands for the fixed point set of *T*; that is, *F*(*T*) = {*x* ∈ *C* : *Tx* = *x*}. A point *p* ∈ *C* is called an* asymptotic fixed point of T* (see [4]) if *C* contains a sequence {*x*_*n*_} which converges weakly to *p* such that lim⁡_*n*→*∞*_||*x*_*n*_ − *Tx*_*n*_|| = 0. We denote by $\hat{F}(T)$ the set of asymptotic fixed points of *T*. A point *p* ∈ *C* is called a* strong asymptotic fixed point of T* if there exists a sequence {*x*_*n*_} in *C* which converges strongly to *p* and lim⁡_*n*→*∞*_||*x*_*n*_ − *Tx*_*n*_|| = 0. We denote the set of all strong asymptotic fixed points of *T* by $\tilde{F}(T)$.

A mapping *T* : *C* → int(dom*f*) with *F*(*T*) ≠ *∅* is called

(i) Bregman quasi-nonexpansive [5] if
  (5) $$\begin{array}{c} D_{f}\left(p,Tx\right)\leq D_{f}\left(p,x\right),\;\forall x\in C,\;\;p\in F\left(T\right); \end{array}$$
(ii) Bregman relatively nonexpansive [5] if
  (6) Df(p,Tx)≤Df(p,x), ∀x∈C,p∈F(T), F^(T)=F(T);
(iii) Bregman firmly nonexpansive [6] if, for all *x*, *y* ∈ *C*,
  (7) 〈∇f(Tx)−∇f(Ty),Tx−Ty〉  ≤〈∇f(x)−∇f(y),Tx−Ty〉;
  or, equivalently,
  (8) Df(Tx,Ty)+Df(Ty,Tx)+Df(Tx,x)+Df(Ty,y)  ≤Df(Tx,y)+Df(Ty,x).
(iv) Bregman weak relatively nonexpansive if
  (9) Df(p,Tx)≤Df(p,x),∀x∈C,p∈F(T), F~(T)=F(T).

**Remark 2**

We observe from the above definitions that every relatively nonexpansive mapping *T* is Bregman relatively nonexpansive mapping with respect to *f*(*x*) = ||*x*||^2^, for all *x* ∈ *E*, where *T* is called* relatively nonexpansive* mapping if the following conditions are satisfied:

(10) ϕ(p,Tx)≤ϕ(p,x), ∀x∈C,p∈F(T), F^(T)=F(T)≠∅.

If *E* = *H*, a real Hilbert space, then relatively nonexpansive mappings are demiclosed quasi-nonexpansive mappings which include the class of nonexpansive mappings with fixed point nonempty.

**Remark 3**

It is shown in [6] that if *T* is Bregman firmly nonexpansive then $\hat{F}(T)=\tilde{F}(T)=F(T)$ and hence it is Bregman relatively nonexpansive provided that the Legendre function *f* is uniformly Fréchet differentiable and bounded on bounded sets of *E*.

**Remark 4**

We observe from the above facts that the class of Bregman weak relatively nonexpansive mappings includes the class of Bregman relatively nonexpansive mappings and hence the class of Bregman firmly nonexpansive mappings. In addition, we also have that every continuous Bregman quasi-nonexpansive is Bregman weak relatively nonexpansive mapping.

The following example by Chen et al. [7] shows that the inclusion is proper.

**Example 5**

Let *E* = *l* ^2^, *f*(*x*) = (1/2)||*x*||^2^, for all *x* ∈ *E*. Let {*x*_*n*_} ⊂ *E* be defined by *x*_0_ = (1,0, 0,…), *x*_1_ = (1,1, 0,0,…), *x*_2_ = (1,0, 1,…)…, *x*_*n*_ = (*ξ*_*n*,1_, *ξ*_*n*,2_,…, *ξ*_*n*,*k*_,…),…, where

(11) $$\begin{array}{c} \xi_{n,k}:=\{\begin{array}{cc} 1, & \text{if}\;\;k=1,\;n+1\\ 0, & \text{otherwise}\;\forall n\geq 1. \end{array} \end{array}$$

Let *T* : *E* → *E* be defined by

(12) $$\begin{array}{c} Tx:=\{\begin{array}{cc} \frac{nx}{n+1}, & \text{if}\;x=x_{n}\\ -x, & \text{if}\;\;x\neq x_{n}\;\forall n\geq 1. \end{array} \end{array}$$

Then, it is shown in [7] that *T* is Bregman weak relatively nonexpansive but not Bregman relatively nonexpansive.

Construction of fixed points of nonexpansive mappings and relatively nonexpansive mappings and their generalizations is an important subject in nonlinear operator theory and its applications, in particular, in image recovery and signal processing (see, e.g., [8–14] and the references therein). Mann [15] and Ishikawa [16] iteration process for approximating fixed point iteration process for nonexpansive mappings and relatively nonexpansive mappings in Hilbert spaces and Banach spaces have been studied extensively by many authors to solve nonlinear operator equations as well as variational inequalities (see, e.g., [15–21]). However, both iteration processes have only* weak convergence* even in Hilbert spaces (see, e.g., [15, 16]). Some attempts to modify the Mann iteration method so that strong convergence is guaranteed have been made.

In 2003, Nakajo and Takahashi [22] proposed the following modification of the Mann iteration method for a nonexpansive mapping *T* : *C* → *C* in a Hilbert space *H*:

(13) $$\begin{array}{c} x_{0}\in C,\;\text{chosen}\;\text{arbitrarily},\\ y_{n}=\alpha_{n}x_{n}+\left(1-\alpha_{n}\right)Tx_{n},\\ C_{n}=\left\{z\in C:||z-y_{n}||\leq ||z-x_{n}||\right\},\\ Q_{n}:=\left\{z\in C:\langle x_{n}-z,x_{n}-x_{0}\rangle \leq 0\right\},\\ x_{n+1}=P_{C_{n}\cap Q_{n}}^{f}\left(x_{0}\right),\;\forall n\geq 0, \end{array}$$

where {*α*_*n*_}⊂[0,1] and *P*_*C*_ denotes the metric projection from *H* onto a closed convex subset *C* of *H*. They proved that the sequence {*x*_*n*_} defined by (13) converges strongly to a fixed point of *T* under some suitable conditions.

In 2005, Matsushita and Takahashi [23] introduced the following modification of the Mann iteration method for a relatively nonexpansive mapping *T* : *C* → *C* in a Banach space *E* as follows:

(14) $$\begin{array}{c} x_{0}\in C,\;\;\text{chosen}\;\;\text{arbitrarily},\\ y_{n}=J^{-1}\left(\alpha_{n}Jx_{n}+\left(1-\alpha_{n}\right)JTx_{n}\right),\\ C_{n}=\left\{z\in C:\phi \left(z,y_{n}\right)\leq \phi \left(z,x_{n}\right)\right\},\\ Q_{n}:=\left\{z\in C:\langle x_{n}-z,Jx_{n}-Jx_{0}\rangle \leq 0\right\},\\ x_{n+1}=\Pi_{C_{n}\cap Q_{n}}^{f}\left(x_{0}\right),\;\forall n\geq 0, \end{array}$$

where {*α*_*n*_}⊂[0,1] and *ϕ*(*y*, *x*) is as shown in Remark 1. They showed that {*x*_*n*_} generated by (14) converges strongly to a fixed point of *T* under some suitable assumptions.

In [24], Reich and Sabach proposed an algorithm for finding a common fixed point of finitely many Bregman firmly nonexpansive mappings *T*_*i*_ : *C* → *C*  (*i* = 1,2,…, *N*) satisfying ⋂_*i*=1_^*N*^ *F*(*T*_*i*_) ≠ *∅* in a reflexive Banach space *E* as follows:

(15) $$\begin{array}{c} x_{0}\in E,\;\;\text{chosen}\;\;\text{arbitrarily},\\ Q_{0}^{i}=E,\\ y_{n}^{i}=T_{i}\left(x_{n}+e_{n}^{i}\right),\\ Q_{n+1}^{i}=\left\{z\in Q_{n}^{i}:\langle \nabla f\left(x_{n}+e_{n}^{i}\right)-\nabla f\left(y_{n}^{i}\right),z-y_{n}^{i}\rangle \leq 0\right\},\\ Q_{n}=\overset{N}{\underset{i=1}{⋂}}Q_{n}^{i},\\ x_{n+1}=P_{Q_{n+1}}^{f}\left(x_{0}\right),\;\forall n\geq 0. \end{array}$$

They proved that, under suitable conditions, the sequence {*x*_*n*_} generated by (15) converges strongly to ⋂_*i*=1_^*N*^ *F*(*T*_*i*_) and applied it to the solution of convex feasibility and equilibrium problems. You may also see [5, 25].

Inspired and motivated by the above works, Chen et al. [7] proposed an algorithm for finding a fixed point of Bregman weak relatively nonexpansive mapping *T*_*i*_ : *C* → *C*  (*i* = 1,2,…, *N*) satisfying *ℱ* : = ⋂_*i*=1_^*N*^ *F*(*T*_*i*_) ≠ *∅* in a reflexive Banach space *E* as follows:

(16) x0∈C, Q0=C,  chosen  arbitrarily,zn=∇f∗(βn∇f(T(xn+en))+(1−βn)∇f(xn+en));yn=∇f∗(αn∇f(T(xn+en))+(1−αn)∇f(zn));Cn={z∈Cn−1∩Qn−1:Df(z,yn)≤Df(z,xn+en)},C0={z∈C:Df(z,y0)≤Df(z,x0)},Qn={z∈Cn−1∩Qn−1:〈∇f(x0)−∇f(xn),z−xn〉≤0},xn+1=PCn∩Qnf(x0), ∀n≥0,

where {*α*_*n*_}, {*β*_*n*_}⊂[0,1] satisfy certain conditions and {*e*_*n*_} is an error sequence in *E* with *e*_*n*_ → 0 as *n* → *∞*. They proved that, under suitable conditions, the sequence {*x*_*n*_} generated by (16) converges strongly to *p* = *P*_*F*(*T*)_^*f*^(*x*_0_), where *P*_*F*(*T*)_^*f*^(*x*_0_) is the Bregman projection of *C* onto *F*(*T*).

Moreover, in [26], Naraghirad and Yao introduced a hybrid iteration algorithm for finding a common fixed point of infinite family of Bregman weak relatively nonexpansive mapping *T*_*i*_ : *C* → *C*  (*i* = 1,2,…) provided that *ℱ* : = ⋂_*i*=1_^*∞*^ *F*(*T*_*i*_) ≠ *∅*, in a reflexive Banach space *E*. They proved that, under suitable conditions, their hybrid iteration algorithm converges strongly to *ℱ*.

**Remark 6**

It is worth mentioning that all the iteration algorithms introduced and used above seem cumbersome and complicated in the sense that at each stage of the iteration computations of the set(s) *C*_*n*_ and/or *Q*_*n*_ are required and the next iterate is taken as the Bergman projection of *x*_0_ on the intersection of *C*_*n*_ and/or *Q*_*n*_. This seems difficult to do in applications.

It is our purpose in this paper to introduce an iterative algorithm for finding a common fixed point of a finite family of Bregman weak relatively nonexpansive mappings in reflexive Banach spaces. We prove strong convergence theorem for the sequence produced by the method. Our scheme does not require computations of the set *C*_*n*_ or *Q*_*n*_ at each stage of iterates. We prove strong convergence theorems for the sequences produced by the method. Our results improve and generalize many known results in the current literature (see, e.g., [7, 23, 24]).

## 2. Preliminaries

Let *f* : *E* → (−*∞*, +*∞*] be a function. For any *x* ∈ Dom⁡(*f*) and *y* ∈ *E*, the right-hand derivative of *f* at *x* in the direction of *y* is defined by *f*°(*x*, *z*) = lim⁡_*t*→0^+^_(*f*(*x* + *ty*) − *f*(*x*))/*t*. The function *f* is said to be* Gâteaux differentiable* at *x* if lim⁡_*t*→0^+^_(*f*(*x* + *ty*) − *f*(*x*))/*t* exists for any *y*. In this case, *f*°(*x*, *y*) coincides with ∇*f*(*x*), the value of the gradient ∇*f* of *f* at *x*. The function *f* is said to be* Gâteaux differentiable* if it is Gâteaux differentiable for any *x* ∈ int(dom*f*). The function *f* is said to be* Fréchet differentiable *at *x* if this limit is attained uniformly in ||*y*|| = 1 and *f* is said to be* uniformly Fréchet differentiable* on a subset *C* of *E* if the limit is attained uniformly for *x* ∈ *C* and ||*y*|| = 1.

Let *x* ∈ int(dom*f*). The subdifferential of *f* at *x* ∈ *E* is the convex set defined by

(17) ∂f(x)={x∗∈E∗:f(x)+〈x∗,y−x〉≤f(y),∀y∈E}.

The* Fenchel conjugate* of *f* is the function f* : E* → (−*∞*, +*∞*] defined by f*(x*) = sup⁡{〈x*, *x*〉−*f*(*x*) : *x* ∈ *E*}.

The function *f* is said to be* essentially smooth* if ∂*f* is both locally bounded and single-valued on its domain. It is called* essentially strictly convex*, if (∂*f*)^−1^ is locally bounded on its domain and *f* is strictly convex on every convex subset of dom⁡∂*f*. *f* is said to be a* Legendre*, if it is both essentially smooth and essentially strictly convex. When the subdifferential of *f* is single-valued, it coincides with the gradient ∂*f* = ∇*f* (see [27]).

For a Legendre function *f* the following properties are known.

- *f* is essentially smooth if and only if f* is essentially strictly convex (see [28, Theorem 5.4]).
- One has (∂*f*)^−1^ = ∂f* (see [29]).
- *f* is Legendre if and only if f* is Legendre (see [28, Corollary 5.5]).
- If *f* is Legendre, then ∇*f* is a bijection satisfying ∇*f* = (∇f*)^−1^, ran∇*f* = dom⁡∇f* = int⁡ dom⁡f*, and ran∇f* = dom⁡∇*f* = int⁡ dom⁡*f* (see [28, Theorem 5.10]).

When *E* is a smooth and strictly convex Banach space, one important and interesting example of Legendre function is *f*(*x*): = (1/*p*)||*x*||^*p*^(1 < *p* < *∞*). In this case the gradient ∇*f* of *f* coincides with the generalized duality mapping of *E*; that is, ∇*f* = *J*_*p*_(1 < *p* < *∞*). In particular, ∇*f* = *I*, the identity mapping in Hilbert spaces.

A function *f* on *E* is* coercive* [30] if the sublevel set of *f* is bounded; equivalently, lim⁡_||*x*||→*∞*_ *f*(*x*) = *∞*. A function *f* on *E* is said to be* strongly coercive* [31] if lim⁡_||*x*||→*∞*_ *f*(*x*)/||*x*|| = *∞*.

Let *B*_*r*_ : = {*z* ∈ *E* : ||*z*|| ≤ *r*}, for all *r* > 0, and *S*_*E*_ = {*x* ∈ *E* : ||*x*|| = 1}. Then, a function *f* : *E* → ℝ is said to be* uniformly convex* on bounded subsets of *E* ([31, pp. 203]) if *ρ*_*r*_(*t*) > 0, for all *r*, *t* > 0, where *ρ*_*r*_ : [0, *∞*)→[0, *∞*] is defined by

(18) ρr(t):=inf⁡x,y∈Br,||x−y||=t,α∈(0,1)((αf(x)+(1−α)f(y)             −f(αx+(1−α)y))            ×(α(1−α))−1),

for all *t* ≥ 0. The function *ρ*_*r*_ is called the* gauge of uniform convexity of f*. The function *f* is also said to be* uniformly smooth* on bounded subsets of *E* if and only if f* is uniformly convex on bounded subsets of *E*.

In the sequel, we will need the following lemmas.

**Lemma 7 (see [26])**

Let *r* > 0 be a constant and let *f* : *E* → ℝ be uniformly convex on bounded subsets of *E*. Then,

(19) $$\begin{array}{c} f\left(\overset{n}{\underset{k=0}{\sum }}\alpha_{k}x_{k}\right)\leq \overset{n}{\underset{k=0}{\sum }}\alpha_{k}f\left(x_{k}\right)-\alpha_{i}\alpha_{j}\rho_{r}\left(||x_{i}-y_{j}||\right), \end{array}$$

for all *i*, *j* ∈ {0,1, 2,…, *n*}, *x*_*k*_ ∈ *Br*, *α*_*k*_ ∈ (0,1), and *k* = 0,1, 2,…, *n*, with ∑_*k*=0_^*n*^ *α*_*k*_ = 1, where *ρ*_*r*_ is the gauge of uniform convexity of *f*.

Let *f* : *E* → (−*∞*, +*∞*] be a Gâteaux differentiable function. The modulus of total convexity of *f* at *x*∈ dom *f* is the function *ν*_*f*_(*x*, .):[0, +*∞*)→[0, +*∞*] defined by

(20) $$\begin{array}{c} \nu_{f}\left(x,t\right):=\inf\left\{D_{f}\left(y,x\right):y\in \mathrm{dom}f,||y-x||=t\right\}. \end{array}$$

The function *f* is called* totally convex at x* if *ν*_*f*_(*x*, *t*) > 0, whenever *t* > 0. The function *f* is called* totally convex* if it is totally convex at any point *x* ∈ int(dom*f*) and is said to be* totally convex on bounded sets* if *ν*_*f*_(*B*, *t*) > 0 for any nonempty bounded subset *B* of *E* and *t* > 0, where the modulus of total convexity of the function *f* on the set *B* is the function *ν*_*f*_ : int(dom*f*)×[0, +*∞*)→[0, +*∞*] defined by

(21) $$\begin{array}{c} \nu_{f}\left(B,t\right):=\inf\left\{V_{f}\left(x,t\right):x\in B\cap \mathrm{dom}f\right\}. \end{array}$$

We know that *f* is totally convex on bounded sets if and only if *f* is uniformly convex on bounded sets (see [32, Theorem 2.10]). The next lemma will be useful in the proof of our main results.

**Lemma 8 (see [27])**

Let *f* : *E* → (−*∞*, +*∞*] be a proper, lower semicontinuous, and convex function; then, for all *z* ∈ *E*, one has

(22) $$\begin{array}{c} D_{f}\left(z,\nabla f^{*}\left(\overset{N}{\underset{i=1}{\sum }}t_{i}\nabla f\left(x_{i}\right)\right)\right)\leq \overset{N}{\underset{i=1}{\sum }}t_{i}D_{f}\left(z,x_{i}\right). \end{array}$$

**Lemma 9 (see [33])**

Let *f* : *E* → ℝ be Gâteaux differentiable on int(dom*f*) such that ∇f* is bounded on bounded subsets of dom⁡f*. Let x* ∈ *X* and {*x*_*n*_} ⊂ *E*. If {*D*_*f*_(*x*, *x*_*n*_)} is bounded, so is the sequence {*x*_*n*_}.

**Lemma 10 (see [32])**

Let *C* be a nonempty, closed, and convex subset of *E*. Let *f* : *E* → ℝ be a Gâteaux differentiable and totally convex function and let *x* ∈ *E*. Then,

- *z* = *P*_*C*_^*f*^(*x*)* if and only if *〈∇*f*(*x*) − ∇*f*(*z*), *y* − *z*〉 ≤ 0, ∀ *y* ∈ *C*;
- *D*_*f*_(*y*, *P*_*C*_^*f*^(*x*)) + *D*_*f*_(*P*_*C*_^*f*^(*x*), *x*) ≤ *D*_*f*_(*y*, *x*),  ∀*y* ∈ *C*.

**Lemma 11 (see [34])**

Let *f* : *E* → (−*∞*, +*∞*] be uniformly Fréchet differentiable and bounded on bounded sets of *E*; then ∇*f* is uniformly continuous on bounded subsets of *E* from the strong topology of *E* to the strong topology of E*.

Let *f* : *E* → ℝ be a Legendre and Gâteaux differentiable function. Following [2, 3], we make use of the function *V*_*f*_ : *E* × E* → [0, +*∞*) associated with *f*, which is defined by

(23) $$\begin{array}{c} V_{f}\left(x,x^{*}\right)=f\left(x\right)-\langle x,x^{*}\rangle +f^{*}\left(x^{*}\right),\;\forall x\in E,\;\;x^{*}\in E^{*}. \end{array}$$

Then, *V*_*f*_ is nonnegative and

(24) $$\begin{array}{c} V_{f}\left(x,x^{*}\right)=D_{f}\left(x,\nabla f^{*}\left(x^{*}\right)\right)\;\forall \;x\in E,\;x^{*}\in E^{*}. \end{array}$$

Moreover, by the subdifferential inequality,

(25) $$\begin{array}{c} V_{f}\left(x,x^{*}\right)+\langle y^{*},\nabla f^{*}\left(x^{*}\right)-x\rangle \leq V_{f}\left(x,x^{*}+y^{*}\right), \end{array}$$

∀*x* ∈ *E*, and x*, y* ∈ E* (see [35]).

## 3. Main Result

In the sequel, we will need the following lemma.

**Lemma 12**

Let *f* : *E* → ℝ be a Legendre function which is bounded, uniformly Fréchet differentiable, and totally convex on bounded subsets of *E*. For *p*, *x*_*i*_ ∈ *E*  (*i* = 1,2,…, *N*) and *α*_*i*_ ∈ [0,1] such that ∑_*i*=0_^*N*^ *α*_*i*_ = 1 one has that

(26) Df(p,∇f∗(α1∇f(x1)+α2∇f(x2)+⋯+αN∇f(xN))) ≤∑i=1NαiDf(p,xi)−αiαjρr∗(||∇f(xi)−∇f(xj)||).

**Proof**

Since *f* is uniformly Fréchet differentiable function we have by Lemma 11 that ∇*f* is uniformly continuous and hence by Theorem 3.6.3 of [31] we get that f* is uniformly convex on bounded subsets of *E*. This, with (24), (23), and Lemma 7, give that

(27) Df(p,∇f∗(α1∇f(x1)+α2∇f(x2)+⋯+αN∇f(xN))) =Vf(p,α1∇f(x1)+α2∇f(x2)+⋯+αN∇f(xN)) =f(p)−〈p,∑i=1Nαi∇f(xi)〉+f∗(∑i=1Nαi∇f(xi)) =f(p)−∑i=1Nαi〈p,∇f(xi)〉+f∗(∑i=1Nαi∇f(xi)) ≤f(p)−∑i=1Nαi〈p,∇f(xi)〉+∑i=1Nαif∗(∇f(xi))  −αiαjρr∗(||∇f(xi)−∇f(xj)||) =∑i=1NαiVf(p,∇f(xi))−αiαjρr∗(||∇f(xi)−∇f(xj)||) =∑i=1NαiDf(p,xi)−αiαjρr∗(||∇f(xi)−∇f(xj)||)

for *i*, *j* ∈ {1,2,…, *N*}. The proof is complete.

We now prove the following theorem.

**Theorem 13**

Let *f* : *E* → ℝ be a strongly coercive Legendre function which is bounded, uniformly Fréchet differentiable, and totally convex on bounded subsets of *E*. Let *C* be a nonempty, closed, and convex subset of int(dom*f*) and let *T*_*i*_ : *C* → *C*, for *i* = 1,2,…, *N*, be a finite family of Bregman weak relatively nonexpansive mappings. Assume that the interior of *F* : = ⋂_*i*=1_^*N*^ *F*(*T*_*i*_) is nonempty. For *x*_0_ ∈ *C* let {*x*_*n*_} be a sequence generated by

(28) $$\begin{array}{c} x_{n+1}=P_{C}^{f}\nabla f^{*}\left(\beta_{0}\nabla f\left(x_{n}\right)+\overset{N}{\underset{i=1}{\sum }}\beta_{i}\nabla f\left(T_{i}x_{n}\right)\right),\;n\geq 0, \end{array}$$

where {*β*_*i*_}_*i*=0_^*N*^ ⊂ [*c*, *d*]⊂(0,1) satisfy lim⁡_*n*→*∞*_ *α*_*n*_ = 0, ∑_*n*=1_^*∞*^ *α*_*n*_ = *∞*, and ∑_*i*=0_^*N*^ *β*_*i*_ = 1. Then, the sequence {*x*_*n*_}_*n*∈*ℕ*_ generated by (28) converges strongly to an element of *F*.

**Proof**

Let *p* ∈ *F*. Then, from (28), Lemma 10, and Lemma 12 we have that

(29) Df(p,xn+1) =Df(p,PCf∇f∗(β0∇f(xn)+∑i=1Nβi∇f(Tixn))) ≤Df(p,∇f∗(β0∇f(xn)+∑i=1Nβi∇f(Tixn))) ≤β0Df(p,xn)+∑i=1NβiDf(p,Tixn)  −β0βiσr(||Tixn−xn||) ≤β0Df(p,xn)+∑i=1NβiDf(p,xn)  −β0βiσr(||Tixn−xn||) ≤Df(p,xn)−β0βiσr(||Tixn−xn||)≤Df(p,xn),

for each *i* ∈ {1,2,…, *N*}, and hence

(30) $$\begin{array}{c} D_{f}\left(p,x_{n+1}\right)\leq D_{f}\left(p,x_{n}\right). \end{array}$$

Therefore, lim⁡_*n*→*∞*_ *D*_*f*_(*p*, *x*_*n*_) exists and hence by Lemma 9 we get that {*x*_*n*_} is bounded. Now, from (2), we also have that

(31) Df(p,xn)−Df(p,xn+1)−Df(xn+1,xn)  =〈∇f(xn)−∇f(xn+1),xn+1−p〉.

This implies that

(32) 〈∇f(xn)−∇f(xn+1),xn+1−p〉+Df(xn+1,xn)  =Df(p,xn)−Df(p,xn+1).

Since the interior of *F* is nonempty, there exists p* ∈ *F* and *r* > 0 such that p* + *rh* ∈ *F*, whenever ||*h*|| ≤ 1. Thus, from (30), we have that

(33) $$\begin{array}{c} D_{f}\left(p^{*}+rh,x_{n+1}\right)\leq D_{f}\left(p^{*}+rh,x_{n}\right). \end{array}$$

Therefore, from (32) and (33), we get that

(34) 0≤〈∇f(xn)−∇f(xn+1),xn+1−(p∗+rh)〉 +Df(xn+1,xn).

Then, this and (32) imply that

(35) r〈∇f(xn)−∇f(xn+1),h〉  ≤〈∇f(xn)−∇f(xn+1),xn+1−p∗〉+Df(xn+1,xn)  =(Df(p∗,xn)−Df(p∗,xn+1))

and hence

(36) 〈∇f(xn)−∇f(xn+1),h〉  ≤1r(Df(p∗,xn)−Df(p∗,xn+1)).

Since *h* with ||*h*|| ≤ 1 is arbitrary, we have

(37) $$\begin{array}{c} ||\nabla f\left(x_{n}\right)-\nabla f\left(x_{n+1}\right)||\leq \frac{1}{r}\left(D_{f}\left(p^{*},x_{n}\right)-D_{f}\left(p^{*},x_{n+1}\right)\right). \end{array}$$

So, if *n* > *m*, then

(38) ||∇f(xm)−∇f(xn)|| =||∇f(xm)−∇f(xm+1)+∇f(xm+1)   −⋯−∇f(xn−1)+∇f(xn−1)−∇f(xn)|| ≤∑i=mn−1||∇f(xi)−∇f(xi+1)|| ≤1r∑i=mn−1(Df(p∗,xi)−Df(p∗,xi+1)) =1r(Df(p∗,xm)−Df(p∗,xn)).

We know that {*ϕ*((p*, *x*_*n*_)} converges. So, {∇*f*(*x*_*n*_)} is a Cauchy sequence. Since E* is complete there exists *y* ∈ E* such that {∇*f*(*x*_*n*_)} converges strongly to *y*. Furthermore, since *f* is Legendre there exists x* ∈ *E* such that *y* = ∇*f*(x*) and hence

(39) $$\begin{array}{c} \nabla f\left(x_{n}\right)⟶\nabla f\left(x^{*}\right)\;\text{as}\;n⟶\infty . \end{array}$$

Now, since *f* is strongly coercive and uniformly convex on bounded subsets of *E*, f* is uniformly Fréchet differentiable and bounded on bounded subsets of *E* (see [31, Prop. 3.6.2]). Thus, applying Lemma 11 we get that *x*_*n*_ = ∇f*(∇*f*(*x*_*n*_)) → x* = ∇f*(∇*f*(x*)), as *n* → *∞*.

Moreover, since {*x*_*n*_} is subset of *C*, which is closed and convex, we have that x* ∈ *C*.

Now, we show that x* ∈ ⋂_*i*=1_^*N*^ *F*(*T*_*i*_). But from (29) we have that

(40) $$\begin{array}{c} \beta_{0}\beta_{i}\sigma_{r}\left(||T_{i}x_{n}-x_{n}||\right)⟶0\;\text{as}\;n⟶\infty \end{array}$$

for each *i* ∈ {1,2,…, *N*}. Then, the property of *σ*_*r*_ implies that ||*T*_*i*_ *x*_*n*_ − *x*_*n*_|| → 0 as *n* → *∞*. From the fact that *T*_*i*_, for each *i* ∈ {1,2,…, *N*}, is Bregman weak relatively nonexpansive we obtain that x* ∈ *F*(*T*_*i*_), for each *i* ∈ {1,2,…, *N*}, and hence x* ∈ ⋂_*i*=1_^*N*^ *F*(*T*_*i*_). This completes the proof.

If, in Theorem 13, every *T*_*i*_ for each *i* ∈ {1,2,…, *N*} is Bregman relatively nonexpansive we have $\hat{F}(T_{i})=\tilde{F}(T_{i})=F(T_{i})$ for each *i* = 1,2,…, *N*. Thus, we get the following corollary.

**Corollary 14**

Let *f* : *E* → ℝ be a strongly coercive Legendre function which is bounded, uniformly Fréchet differentiable, and totally convex on bounded subsets of *E*. Let *C* be a nonempty, closed, and convex subset of int(dom*f*) and let *T*_*i*_ : *C* → *C*, for *i* = 1,2,…, *N*, be a finite family of Bregman relatively nonexpansive mappings. Assume that the interior of *ℱ* : = ⋂_*i*=1_^*N*^ *F*(*T*_*i*_) is nonempty. For *x*_0_ ∈ *C* let {*x*_*n*_} be a sequence generated by (28). Then, the sequence {*x*_*n*_}_*n*∈*ℕ*_ converges strongly to an element of *ℱ*.

If, in Theorem 13, we take *T*_*i*_, for each *i* = 1,2,…, *N*, to be continuous Bregman quasi-nonexpansive mappings, then we have $\tilde{F}(T_{i})=F(T_{i})$ for each *i* = 1,2,…, *N*. Thus, we have the following corollary.

**Corollary 15**

Let *f* : *E* → ℝ be a strongly coercive Legendre function which is bounded, uniformly Fréchet differentiable, and totally convex on bounded subsets of *E*. Let *C* be a nonempty, closed, and convex subset of int(dom*f*) and let *T*_*i*_ : *C* → *C*, for *i* = 1,2,…, *N*, be a finite family of continuous Bregman quasi-nonexpansive mappings. Assume that the interior of *ℱ* : = ⋂_*i*=1_^*N*^ *F*(*T*_*i*_) is nonempty. For *x*_0_ ∈ *C* let {*x*_*n*_} be a sequence generated by (28). Then, the sequence {*x*_*n*_}_*n*∈*ℕ*_ converges strongly to an element of *ℱ*.

If, in Theorem 13, we take *N* = 1, then we have the following corollary.

**Corollary 16**

Let *f* : *E* → ℝ be a strongly coercive Legendre function which is bounded, uniformly Fréchet differentiable, and totally convex on bounded subsets of *E*. Let *C* be a nonempty, closed, and convex subset of int(dom*f*) and let *T* : *C* → *C* be a finite family of Bregman relatively nonexpansive mappings. Assume that the interior of *F*(*T*) is nonempty. For *x*_0_ ∈ *C* let {*x*_*n*_} be a sequence generated by

(41) $$\begin{array}{c} x_{n+1}=P_{C}^{f}\nabla f^{*}\left(\beta \nabla f\left(x_{n}\right)+\left(1-\beta \right)\nabla f\left(Tx_{n}\right)\right),\;n\geq 0, \end{array}$$

where *β* ∈ (0,1), {*α*_*n*_}⊂(0,1) satisfy lim⁡_*n*→*∞*_ *α*_*n*_ = 0 and ∑_*n*=1_^*∞*^ *α*_*n*_ = *∞*. Then, the sequence {*x*_*n*_}_*n*∈*ℕ*_ generated by (41) converges strongly to an element of *ℱ*.

**Remark 17**

Our results are new even if the convex function *f* is chosen to be *f*(*x*) = (1/*p*)||*x*||^*p*^  (1 < *p* < *∞*) in uniformly smooth and uniformly convex spaces.

**Remark 18**

Our theorems improve and unify most of the results that have been proved for these important classes of nonlinear operators. In particular, Theorem 3.2 extends Theorem 3.1 of [23], Theorem 1 of [24], and Theorem 3.1 of [7] in the sense that either our theorem is applicable to a more general class of a finite family of Bregman weak relatively nonexpansive mappings or our scheme does not require the computation of *C*_*n*_ or *Q*_*n*_ for each *n* ≥ 0 provided that the interior of *ℱ* is nonempty.

Moreover, we observe that Theorem 3.2 extends Theorem 3.1 of [26] in the sense that our scheme does not require the computations of *C*_*n*_ for each *n* ≥ 0 when we consider finite family of Bregman weak relatively nonexpansive mappings and the interior of *ℱ* is nonempty.
